# Comparative analysis of endophyte diversity of *Dendrobium officinale* lived on rock and tree

**DOI:** 10.5511/plantbiotechnology.23.0208a

**Published:** 2023-06-25

**Authors:** Xiaolan Li, Huan Hu, Qunli Ren, Miao Wang, Yimei Du, Yuqi He, Qian Wang

**Affiliations:** 1 Microbial Resources and Drug Development Key Laboratory of Guizhou Tertiary Institution, Life Sciences Institute, School of Stomatology, Zunyi Medical University, Zunyi 563000, China;; 2 School of Pharmacy, Zunyi Medical University, Zunyi 563000, China; 3 Key Laboratory of Basic Pharmacology of Ministry of Education and Joint International Research Laboratory of Ethnomedicine of Ministry of Education, Zunyi Medical University, Zunyi 563000, China

**Keywords:** *Dendrobium officinale*, diversity, endophytic bacteria, endophytic fungi, epiphytic substrates

## Abstract

*Dendrobium officinale* usually lives on rock or tree, but their endophyte diversity has not yet been fully revealed? In this study, high-throughput sequencing technology was used to investigate the endophyte diversity of the roots of *D. officinale* lived on tree (Group 1–3, arboreal type) and rock (Group 4, lithophytic type). The results showed that their composition of endophytic fungi and bacteria were similar at phylum level, while their relative abundance were different. Their taxa composition and abundance of endophytes differed significantly among groups at the genus level. *Alpha* diversity of endophytic fungi of lithophytic type was higher than those from arboreal type, while there was no advantage in endophytic bacteria. *Beta* diversity revealed that the endophytic fungi tended to cluster in each group, but the endophytic bacteria were dispersed among the groups. LEfSe analysis found that the numbers of predicted endophyte biomarkers of lithophytic type were more than arboreal types at genus level, and the biomarkers varied among groups. Microbial network analysis revealed similarities and differences in the taxa composition and abundance of shared and special endophytes in each group. These results suggested that the root endophytes of lithophytic and arboreal *D. officinale* differed in diversity.

## Introduction

*Dendrobium officinale* Kimura et Migo belongs to the *Dendrobium* (Orchidaceae), which is one of the best-known traditional and precious Chinese medicinal materials, and contains various of active ingredients such as dendrobium polysaccharide, dendrobium alkaloid, these ingredients are effective in protecting diabetic kidney damage (Chang et al. 2019), inhibiting the proliferation of liver cancer cells and inducing their apoptosis (Guo et al. 2019), etc. The self-propagation ability of *D. officinale* is quite weak in nature, furthermore, its wild resources was increasingly depleted due to long-term over-exploitation, so nowadays its medicinal materials are mainly derived from artificial cultivation (Zhou et al. 2018). Therefore, the research on the favorable factors for the survival of *D. officinale* will contribute to its mass propagation and accumulation of medicinal components in its artificial cultivation. *D. officinale* is usually epiphytic on the stems of broad-leaved forest trees or rock surfaces (Cheng et al. 2004; Lu and Chai 2019). To adapt to this habitat, its aerial roots develop a special structure—Velamen radicum, a spongy, usually multiple epidermis of the roots, which at maturity consists of dead cells. The functions of Velamen radicum are efficient water and nutrient uptake, nutrient retention, reduction of water loss, mechanical protection, or the avoidance of overheating (Hauber et al. 2020; Oliveira et al. 2019; Zhang et al. 2018; Zotz and Winkler 2013).

Moreover, endophytes are widely colonized in plant tissues and coexist with its host, which contain two major categories, endophytic fungi and endophytic bacteria, and play an important role in plant growth, stress resistance and development (Gouda et al. 2016; Khare et al. 2018; Manzotti et al. 2020). Especially in orchid species, they must rely on endophytes to achieve seed germination, growth and stress adaptation due to lack of endosperm (Pant et al. 2017). Endophytic fungi were beneficial to *D. officinale* with functions such as promoting growth (Chen et al. 2010). However, there are relatively few studies on endophytic bacteria of *D. officinale*. Numerous studies found that, endophytic bacteria are beneficial to plants and can enhance plant growth under normal and stress conditions. They can directly improve plant nutrient uptake and modulate growth and stress related phytohormones, and indirectly improve plant health by utilizing antibiotics, hydrolases, nutrient limitations to target pests and pathogens and initiate plant defenses (Afzal et al. 2019). It is evident that the presence of Velamen radicum and endophytes is an important favorable condition that help *D. officinale* to grow on relatively harsh habitats such as stone surfaces and tree trunks, and its root endophyte resources may be of high value for exploitation and utilization.

Some beneficial endophytes have a wide host range and can be used as biological inoculants to develop sustainable artificial cultivation (Lally et al. 2017), and can facilitate the study of related assisting mechanisms of plant endophytes, of which the diversity analysis of endophytes is the basis. The diversity of endophytes is affected by many factors, among which the plant’s habitat factor is one of the most important factors. Therefore, studying the diversity of *D. officinale* root endophytes in different habitats is very beneficial to mining its beneficial endophytes. With the development of microbial isolation, culture technology and high-throughput sequencing technology, the research on plant endophytes has become a hot spot in the world. The high-throughput sequencing methods enable identification and relative quantification of community members and offer new insights into microbial community ecology, and these methods are currently taking over as the primary tool to assess plant-associated endophytes communities (Lindahl et al. 2013). This study therefore focuses on using high-throughput sequencing technology to investigate the root endophytes diversity of *D. officinale* in different habitats (lithophytic and arboreal) in the Southwest Guizhou planting base, laying a foundation for the subsequent excavation of beneficial endophytes.

## Materials and methods

### Root sample collection and surface disinfection of *D. officinale*

The whole plants of *D. officinale* were collected from the trunks of *Albizia julibrissin* Durazz, *Cyclobalanopsis myrsinifolia* (Blume) Oersted and sedimentary rocks from June 18th to 20th, 2019. The sampling locations were planting bases of *D. officinale* in Zhegui Village, Qifeng Street, Anlong County, Guizhou Province (E105°24′54″, N24°58′37″, Group 1 with 9 samples and Group 2 with 8 samples), and Zerong Township, Xingyi City, Guizhou Province (E104°57′14″, N24°54′19″, Group 3 with 5 samples and Group 4 with 7 samples), respectively. The living plants with complete root system were packed in kraft paper bags and frozen with dry ice, then transported to the laboratory. The roots of *D. officinale* were rinsed with running water, absorbed the residual water with sterile filter paper. Three undamaged roots per plant without velamen removal were selected and cut to 2 cm root segments, then rinsed 3 times with sterile distilled water, disinfected their surface with 75% ethanol for 20 s, and rinsed 3 times with sterile distilled water. After absorbed the water with the sterile filter paper, they were stored in the refrigerator at −80°C for future usage. The sample information was shown in Table 1. Before the samples were used for sequencing, the surface-sterilized samples were rinsed 3 times with sterile distilled water, and the sterile distilled water from the third rinse was used as samples for qPCR detection to confirm the complete surface sterilization of the root.

**Table 1:** Table 1. Sample information.

Groups	Number of samples	Epiphytic substrate	Annual average temperature (°C)	Annual average precipitation (mm)	Annual average relative humidity (%)	Altitude (m)	Sun exposure	Sampling location
Group 1	9	*A. julibrissin*	15.3	1,195.4	81	975	shaded	XCXS Planting Base, Anlong, Guizhou Province (E105°24′54″, N24°58′37″)
Group 2	8	*C. myrsinifolia*	15.3	1,195.4	81	975	shaded	
Group 3	5	*C. myrsinifolia*	17.5	1,325.9	80	1,206	shaded	SCG Planting Base, Zerong, Guizhou Province (E104°57′14″, N24°54′19″)
Group 4	7	Sedimentary rock	17.5	1,325.9	80	1,206	shaded	

### Amplification of target DNA fragments

#### Genomic DNA extraction

Since the DNeasy PowerSoil Kit (QIAGEN) for DNA extraction facilitated microbial diversity analysis (Mattei et al. 2019; Pearman et al. 2020), the Kit was used to extract total DNA once the root tissues were frozen in liquid nitrogen and further homogenized. The concentration and purity of total DNA were determined using the NanoDrop Spectrophotometer. The extracted DNA was diluted with sterile ultrapure water to 10 ng·µl^−1^ and stored in the refrigerator at −80°C for later use.

#### PCR amplification, detection and purification

The primers for the 16S rRNA V4 region are 515F (5′-GTGCCAGCMGCCGCGGTAA-3′) and 806R (5′-GGACTACHVGGGTWTCTAAT-3′) (The amplification products of bacteria were isolated using the patented technology NO. CN 106282165 B) (Caporaso et al. 2011; Liu W et al. 2017), and universal primers of ITS II region are ITS3_KYO2 (5′-GATGAAGAACGYAGYRAA-3′) and ITS4 (5′-TCCTCCGCTTATTGAT ATGC-3′) (Toju et al. 2012). The diluted genomic DNA was used as a template, according to the selection of the sequencing region, using specific primers with barcode for PCR amplification. The 25 µl PCR system included 1×PCR buffer, 1.5 mM MgCl_2_, 0.4 µM dNTPs, 1.0 µM forward and reverse primers, 0.5 U KOD-Plus-Neo enzyme (TOYOBO) and 10 ng DNA template. PCR reaction program: 94°C pre-denaturation 1 min, 30 cycles (denaturation 94°C 20 s, annealing 54°C (16S rRNA V4)/48°C (ITS2) 30 s and extension 72°C 30 s), and finally 72°C extension 5 min. Each sample was repeated three times. After PCR amplifications were completed, the PCR products of the same sample were mixed and detected by 2% agarose gel electrophoresis. The PCR products with bright bands between 200 and 400 bp were selected and recovered with the QIA quick Gel Extraction Kit (QIAGEN). Qubit@ 2.0 Fluorometer (Thermo Scientific) was used for quantitative detection. The PCR products of different samples were mixed in equimolar amounts for later use.

#### Library construction and sequencing

Library was constructed and tagged according to the instruction manual of TruSeq DNA PCR-Free Sample Prep Kit. After the library was qualified by Qubit@ 2.0 Fluorometer (Thermo Scientific) and Agilent Bioanalyzer 2100 system, Hiseq 2500 platform PE250 and Hiseq Rapid SBS Kit v2 (Illumina) were used for paired-end sequencing. The samples were sent to Chengdu Luoning Biotechnology Co., Ltd. for high-throughput sequencing of 16S rRNA V4 region and ITS II region.

### Analysis of sequencing data

#### Sequence assembly, quality control, OTU clustering and annotation

The sequences were analyzed according to Usearch (http://drive5.com/uparse/) and QIIME (Caporaso et al. 2010b) pipeline. Paired-end reads from the original DNA fragments were merged using FLASH (Magoč and Salzberg 2011). Then sequences were assigned to each sample according to the unique barcode. After the tag sequences were cut off, the effective sequences were used for quality control by Trimmomatic (Bolger et al. 2014) and Usearch, and the chimera were removed by the Uchime algorithm (Robert et al. 2011). The sequences were analyzed by UPARSE algorithm according to Usearch (Edgar 2013) for OTU clustering at a consistency level of 97%. The sequence with the highest frequency in each OTU was selected as the representative sequence of OTU, and annotation analysis was performed using UCLUST classification method (Edgar 2010) with SILVA database (http://www.arb-silva.de/) for bacteria (Quast et al. 2013) and UNITE database (https://unite.ut.ee/) for fungi (Nilsson et al. 2019).

#### Phylogenetic tree analysis

Representative sequences were aligned using PyNAST12 (Caporaso et al. 2010a) embedded in QIIME (Caporaso et al. 2010b). After quality checking, phylogenetic trees were reconstructed based on maximum likelihood–approximation method using the generalised time-reversible (GTR) model in FastTree (Price et al. 2010).

#### Statistics

In case of the influences of sequencing depth on community diversity, the OTU table was rarified to make all samples holding the same sequence number (9385). All data analyses were performed using R (Team 2014) or Python (https://www.python.org/). The random seed number was fixed at 1234. Phylogenetic diversity (Faith’s PD) was calculated using Picante (Faith 1992; Kembel et al. 2010). Weighted and Unweighted Unifrac distances were calculated in GUniFrac (Chen 2012). Other alpha- and beta-diversity metrics were calculated in Vegan (Oksanen et al. 2016). Rarefaction curves were generated based on these three metrics (Kembel et al. 2010). Kruskal–Wallis rank sum test was performed to show the significance of the difference on alpha diversity and taxa among different groups (Acar and Sun 2013). Principal component analysis (PCA) was applied to reduce the dimensions of original community data (Abdi and Williams 2010). Hierarchical cluster analysis was done using R function hclust (Langfelder and Horvath 2012). To identify if there were significant differences among different groups, permutational multivariate analysis of variance (PERMANOVA) was performed based on the dissimilarity substrate (Chen and Zhang 2022). Permutational analysis of multivariate dispersions (PERMDISP) was used for the analysis of multivariate homogeneity of group dispersions using the Vegan function betadisper (Anderson et al. 2008). Random Forests analysis was applied to obtain the important indicator taxa using random Forest package with 1,000 trees and all default settings (Nguyen et al. 2021). We used distance-based redundancy analysis (dbRDA) to partition variation in beta-diversity into fractions explained by environmental variables (Legendre and Anderson 1999). The functions “dbrda”, “anova.cca”, “vif.cca” and “ordiR2step” in R package Vegan were used to perform the model construction, variables and axes significance permutation test, variance inflation factor analysis and forward selection (Oksanen et al. 2016). Statistical comparisons of mean values for paired data and those for data more than two sets were done with wilcox.test function (Johnson et al. 2009) and kruskal.test function (Doane et al. 2020) in R, respectively. The level of statistical significance was set at *p*<0.05. Random Forest analyses were done using R random Forest package (Stephan et al. 2015). LEfSe analysis were done using Python LEfSe package (Segata et al. 2011). Network analysis was performed on Tutoolsplatform (http://www.cloudtutu.com) and Cytoscape 3.8.2.

## Results

### Sequencing data and the number of OTUs

After sequencing endophytic fungus ITS II region, we got 28,698 to 38,035 high quality sequences with an average length of 310–384 bp. The OTUs of endophytic fungi from Group 1 to Group 4 were 602, 523, 360 and 624, respectively. The number of high-quality sequences obtained by sequencing 16S rRNA V4 region of endophytic bacteria, which was 29,919 to 37,832 with an average length of 312–327 bp. The OTUs of endophytic bacteria from Group 1 to Group 4 were 1,479, 1,299, 1,071, and 1,462, respectively. The sequence information was listed in Table 2. The sequence has been deposited to the National Center for Microbiology Science in China (accession number: NMDCX0000108).

**Table 2:** Table 2. Sequence information of endophytic fungi and bacteria from the roots of *D. officinale.*

Groups	Effective sequences		OTUs	
	ITS	16S rRNA	ITS	16S rRNA
Group 1	28,757–37,169	30,242–37,832	602	1,479
Group 2	28,698–38,024	29,942–36,551	523	1,299
Group 3	29,262–37,203	31,449–37,739	360	1,071
Group 4	28,698–38,035	29,919–37,832	624	1,462

### Endophytes community composition

**Figure 1:**
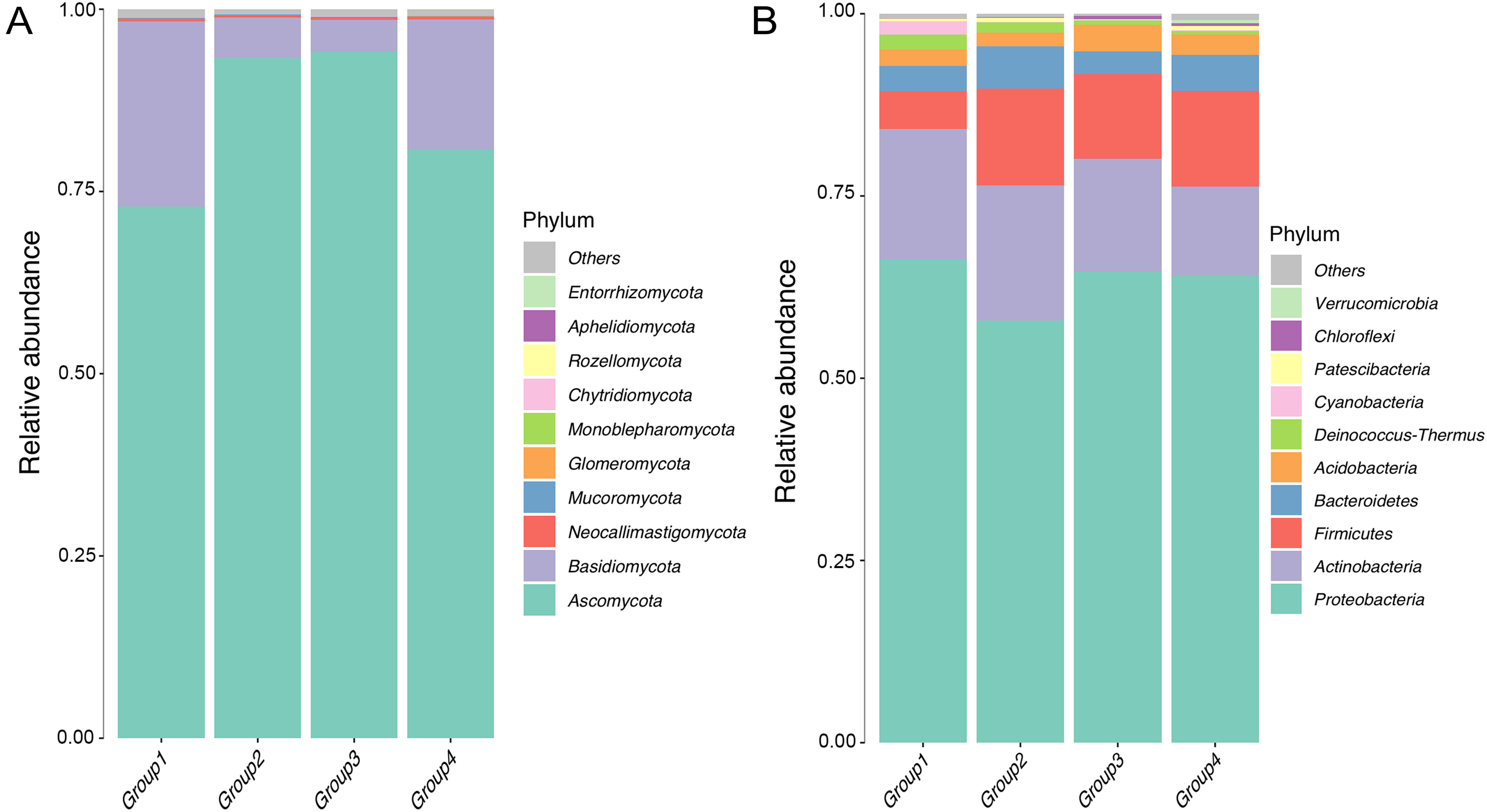
Figure 1. Barplots of relative abundance of endophytes in *D. officinale* root at phylum level (mean of each group). (A) is barplot of relative abundance of endophytic fungi at phylum level, (B) is barplot of relative abundance of endophytic bacteria at phylum level.

To explore the average abundance of main taxa of each group and the degree of similarity between groups, the data of the groups were averaged and displayed in stacked bar plots (Figure 1). At the phylum level, more than 90% of the endophytic fungi in the roots of *D. officinale* living on different substrates were Basidiomycota (18 genera) and Ascomycota (70 genera), in which the Ascomycota accounted for more than 70%. In Group 2 and Group 3 (living on *Cyclobalanopsis myrsinifolia*), the proportions of Basidiomycetes and Ascomycetes were similar. However, there was a notorious difference in the community composition of endophytic fungi between Group 1 and Group 4 (from the roots of *D. officinale* living on the trunk of *Albizia julibrissin* and sedimentary rocks, respectively). Among the top ten phylums of endophytic fungi (97 genera altogether), the other endophytic fungi accounted for a relatively small proportion, including Aphelidiomycota, Chytridiomycota, Entorrhizomycota, Glomeromycota, Monoblepharomycota, Mucoromycota, Neocallimastigomycota, Rozellomycota. The species and relative abundance of endophytic fungi in the roots of *D. officinale* living on the trunk of *Cyclobalanopsis myrsinifolia* (Blume) Oersted (Group 2 and Group 3) were similar, while the relative abundance of endophytic fungi varied greatly between the samples from different tree species (Figure 1A). At the phylum level, the dominant endophytic bacteria in the roots of *D. officinale* mainly included Proteobacteria, Actinobacteria, Firmicutes, Bacteroidetes, Acidobacteria and Patescibacteria, among which the dominant groups were Proteobacteria, Actinobacteria and Firmicutes, their relative abundances were 57–66%, 12–18%, and 5–13%, respectively (Figure 1B). In general, at the phylum level, the community composition of endophytic fungi and endophytic bacteria in the roots of *D. officinale* living on the trees and rocks were similar, but their relative abundance varied greatly depending on the different epiphytic substrate.

**Figure 2:**
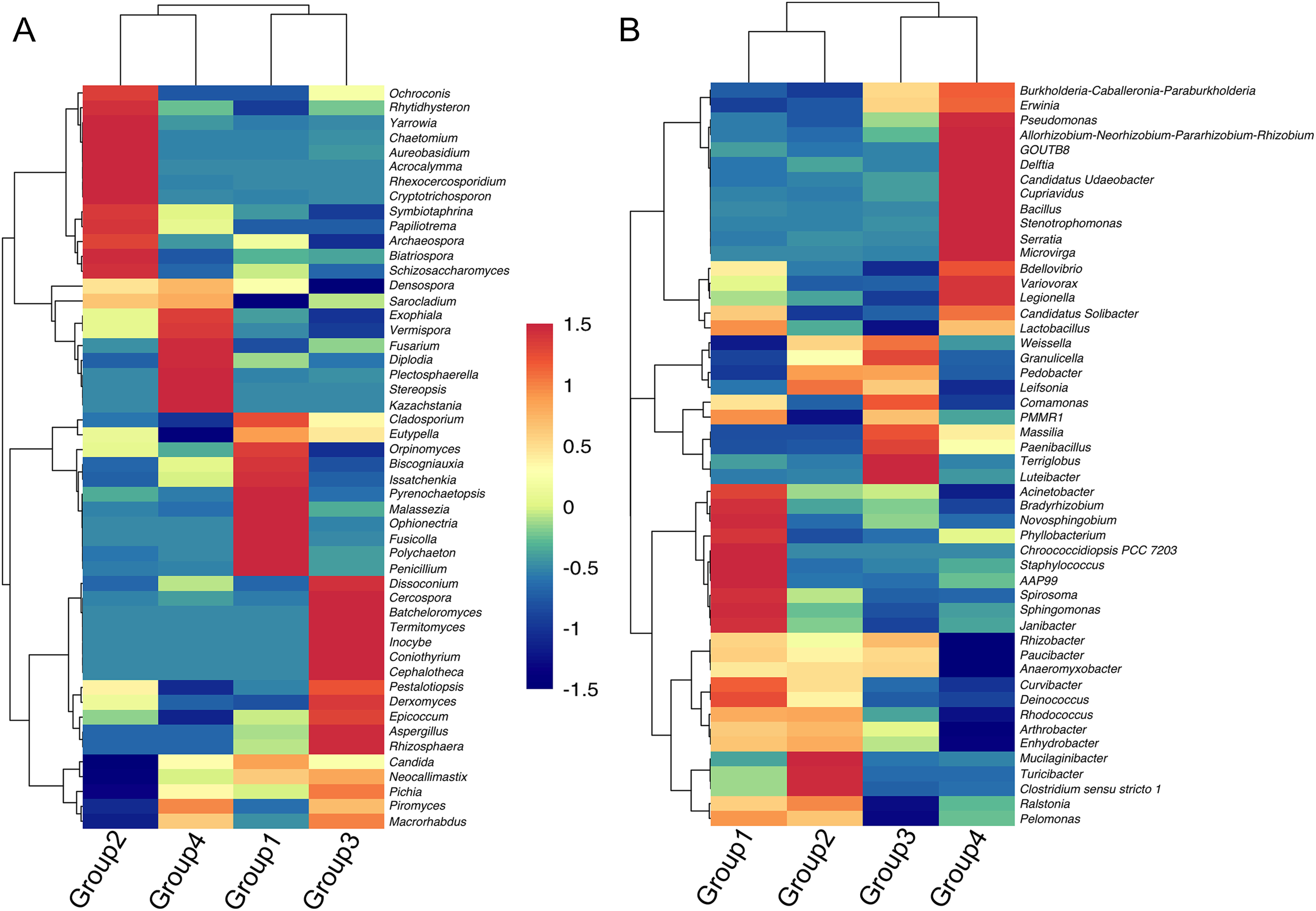
Figure 2. Heatmaps of top 50 endophytes genus in *D. officinale* root. (A) heatmap of endophytic fungi, (B) heatmap of endophytic bacteria.

The heat map was used to analyze the similarity of endophytes at the genus level (Figure 2). For the top 50 endophytes in each group, their abundance was different, and each group had its own unique dominant endophyte genus. In each group, the endophytic fungi with high abundance showing higher similarity and tended to cluster into one group, and the clustering of endophytic bacteria was relatively discrete in each group except Group 4. At the genus level, the dominant endophytes in the root of *D. officinale* varied significantly according to different planting bases. Besides, the abundance of endophytes was also differed from different epiphytic substrates. For endophytic fungi, the genera with high abundance were clustered into a branch, there were 11 dominant genera in Group 1, including *Polychaeton*, *Penicillium* and *Cladosporium*; 13 dominant genera in Group 2, including *Yarrowia*, *Rhexocercosporidium*, and *Chaetomium*; 9 dominant genera in Group 3, including *Aspergillus*, *Cercospora*, and *Termitomyces*; 7 dominant genera in Group 4, including *Diplodia*, *Exophiala*, and *Fusarium*. For endophytic bacteria, they were 12 dominant genera in Group 1, including *Spirosoma* and *Staphylococcus*, 3 dominant genera in Group 2, including *Mucilaginibacter*, *Turicibacter*, and *Clostridium sensu stricto* 1, 6 dominant genera in Group 3, including *Terriglobus* and *Luteibacter*, 12 dominant genera in Group 4, including *Pseudomonas*, *Bacillus*, and *Delftia*, respectively. In general, the dominant endophytes at genus level in the roots of *D. officinale* were different and changed with different growth substrates and regions.

### The abundance, evenness and diversity of the microbial community

**Figure 3:**
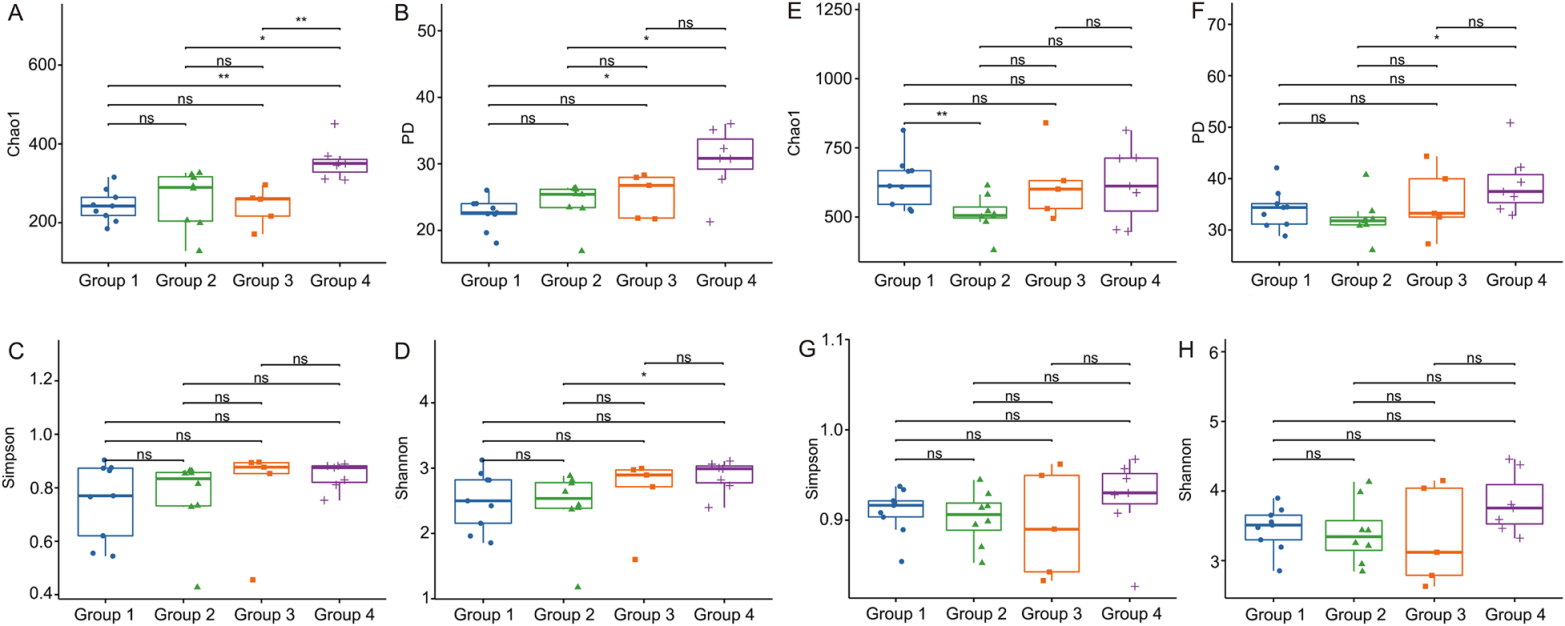
Figure 3. Boxplots of Alpha diversity of endophytic fungi and bacteria in *D. officinale* root. (A–D) are boxplots of Chao1, Faith’s PD, Simpson and Shannon index of endophytic fungi, respectively. (E–H) are boxplots of Chao1, Faith’s PD, Simpson and Shannon index of endophytic bacteria, respectively. *, *p*<0.05; **, *p*<0.01, “ns” indicates no significant difference.

The *alpha* diversity reflected abundance, evenness and diversity of the microbial community. This study analyzed Chao1, Faith’s PD, Simpson, and Shannon’s diversity indexes (Figure 3). In endophytic fungi (Figure 3A–D), the highest Chao1 of endophytic fungi was in Group 4, and it were significantly different from that in Group 1 (*p*<0.01), Group 3 (*p*<0.01) and Group 2 (*p*<0.05); there was no significant difference between Group 1–3. The highest Faith’s PD index was in Group 4, which is significantly different from Group 1 and Group 2 (*p*<0.05), and there was no significant difference between the other Groups. The Simpson index showed no significant difference between groups. The highest Shannon index was in Group 4 and it was significantly different from Group 2 (*p*<0.05). In endophytic bacteria (Figure 3E–H), the Chao1 index was significantly different between Group 1 and Group 2 (*p*<0.01), and the faith’s PD index showed significant difference between Group 4 and Group 2 (*p*<0.05), however, for the indexes of Chao1, faith’s PD, Simpson and Shannon, there was no significant difference among the other groups. The results showed that the roots of *D. officinale* living on rock had higher alpha diversity of endophytic fungi than that living on tree trunks, while the alpha diversity of endophytic bacteria in the roots of *D. officinale* living on rock didn’t have a distinct advantage.

### The differences and variation between Groups

**Figure 4:**
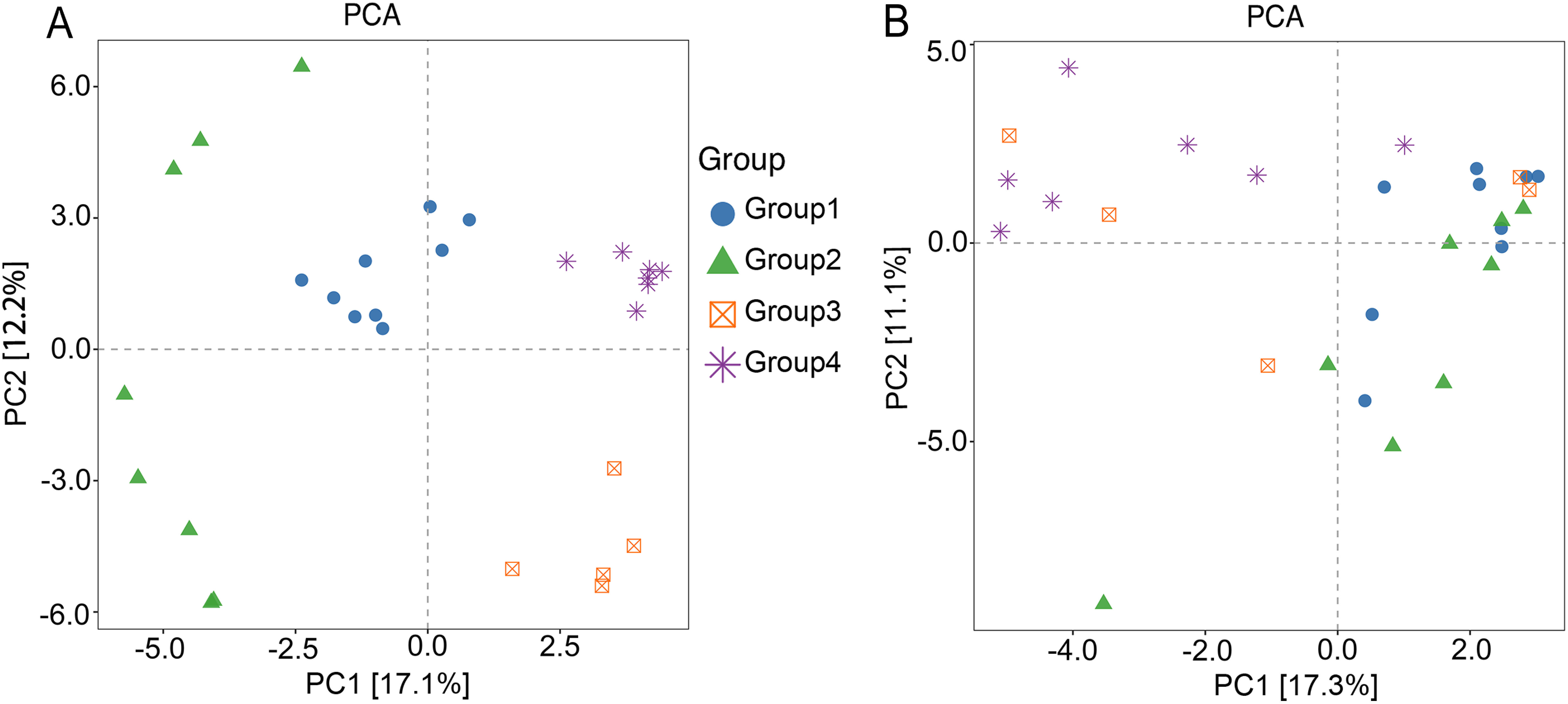
Figure 4. PCA analysis for endophytes in *D. officinale* root. (A) is PCA analysis for endophytic fungi, (B) is PCA analysis for endophytic bacteria.

Principal component analysis (PCA) of *beta* diversity was used to explore the differences and its variation between Groups. As showed in Figure 4A, the endophytic fungi relatively clustered in Group 1, 3, and 4, with Group 4 being the most clustered, and *p* values of their PERMANOVA and PERMDISP were 0.001 and 0.094 (Table 3), respectively. Figure 4B showed that the endophytic bacteria in each group couldn’t cluster, and the *p* values of their PERMANOVA and PERMDISP were 0.001 and 0.152 (Table 3), respectively. The information indicated that the taxa compositions of endophytic fungi and endophytic bacteria were significantly different. The results showed that the communities of endophytic fungi in *D. officinale* root tended to cluster in each group, but the communities of endophytic bacteria were dispersed among the groups, which may be related to the combined effect of various factors, such as different epiphytic substrates, altitude, precipitation, and temperature conditions. In the same habitat, different epiphytic substrates may have a greater impact on endophytic fungi, such as Group 3 and Group 4; but for endophytic bacteria, the situation is more complicated, and it is difficult to distinguish between them, such as Group 3 and Group 4. In different habitats, other environmental factors may have a greater impact on endophytic fungi, such as Group 2 and Group 3; for endophytic bacteria, it may be affected by various environmental factors in the habitat and it is difficult to distinguish the main influencing factors.

**Table 3:** Table 3. The results of permutational multivariate analysis of variance (PERMANOVA) and permutational analysis of multivariate dispersions (PERMDISP).

Endophytes	PERMANOVA			PERMDISP		
	R2	*p*-value	Significance	Mean Sq	*p*-value	Significance
Fungi	0.395	0.001	**	0.068	0.094	
Bacteria	0.213	0.001	**	0.020	0.152	

Significance: **, *p*<0.01; *, *p*<0.05. R2, Variation (R2), variance contribution. Mean Sq, mean squared error.

### The predicted biomarkers and networks of endophyte

**Figure 5:**
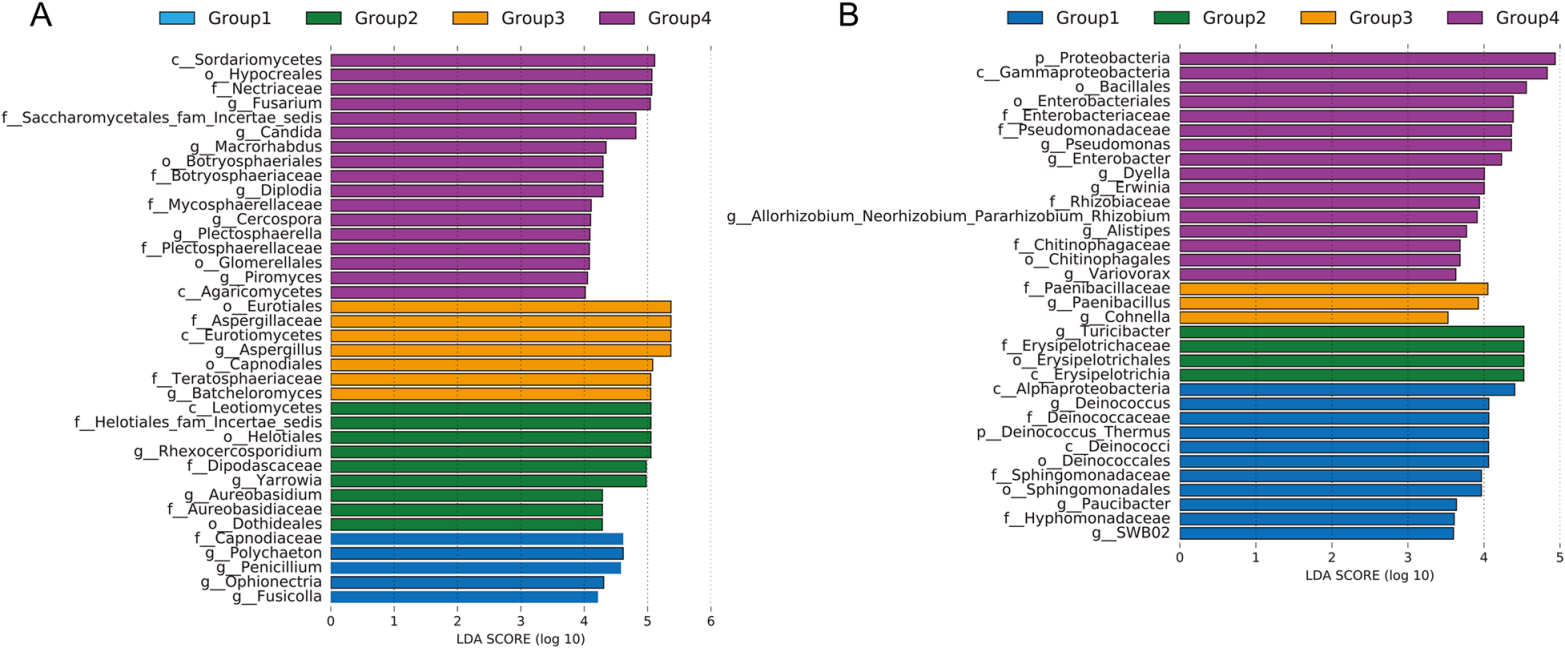
Figure 5. LDA values of endophytes in *D. officinale* root. (A) endophytic fungi, threshold of LDA score is 4; (B) endophytic bacteria, threshold of LDA score is 3.5.

LEfSe was used to predict biomarkers of endophyte in each group. From Figure 5, it can be found that each group has its own specific biomarkers. Among endophytic fungi, the biomarkers of Group 1 were *Polychaeton*, *Capnodiaceae*, *Penicillium*, etc.; the biomarkers of Group 2 were *Leotiomycetes*, *Rhexocercosporidium*, *Helotiales*, etc.; the biomarkers of Group 3 were *Eurotiomycetes*, *Aspergillaceae*, *Eurotiales*, etc.; the biomarkers of Group 4 were *Fusarium*, *Hypocreales*, *Sordariomycetes*, etc. (Figure 5A). Among endophytic bacteria, the biomarkers of Group 1 were *Alphaproteobacteria*, etc.; the biomarkers of Group 2 were *Turicibacter*, etc.; the biomarkers of Group 3 were *Paenibacillaceae*, etc.; the biomarkers of Group 4 were *Rhizobiaceae*, *Pseudomonas*, etc. (Figure 5B). LDA score of these biomarkers was all greater than 3.5 (*p*<0.05), indicating that each group has its own dominant endophytic fungi and endophytic bacteria. Moreover, Cladogram information on these biomarkers was in Supplementary Files.

**Figure 6:**
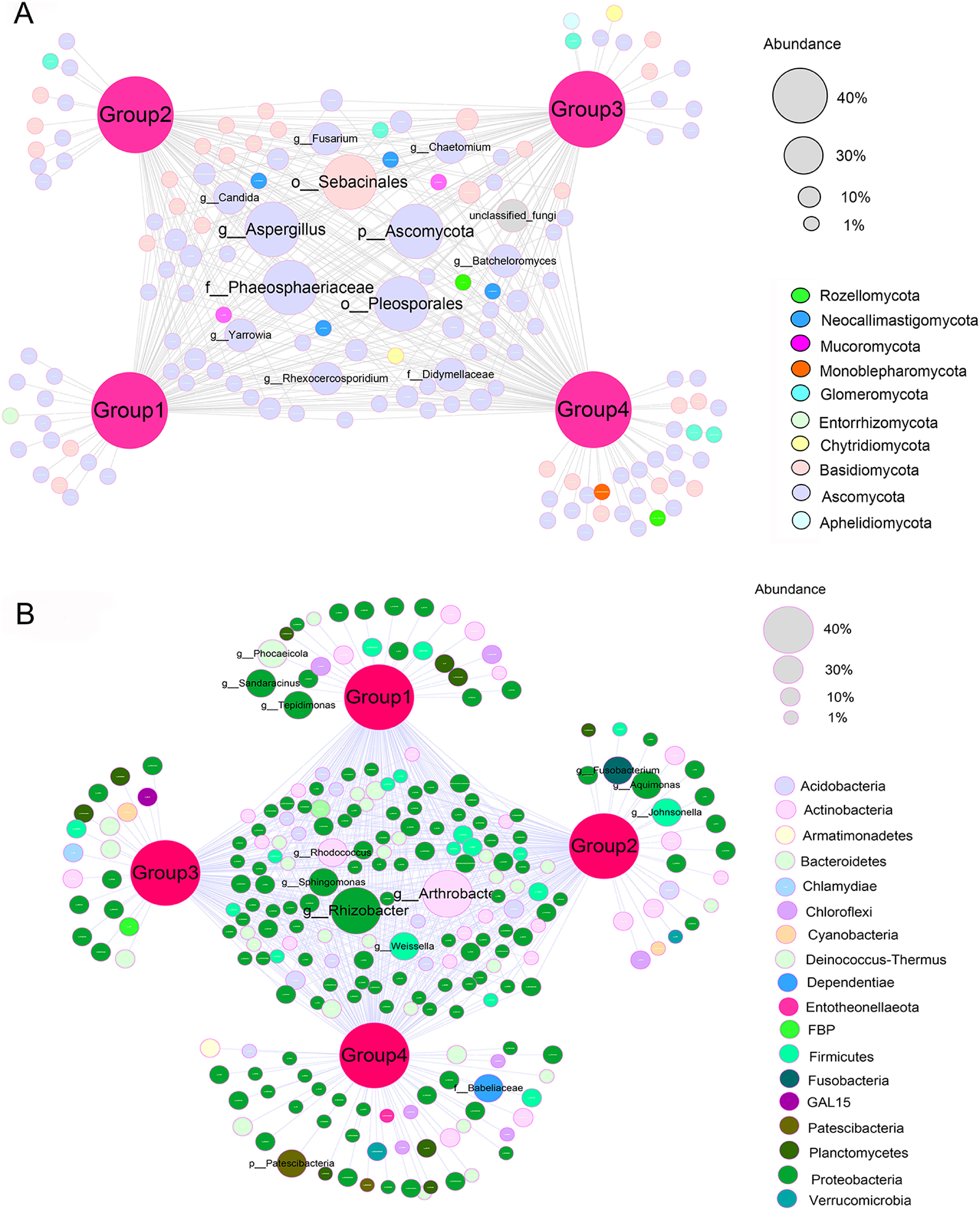
Figure 6. Networks of endophytes in *D. officinale* root.

The results of further network analysis were shown in Figure 6. The abundance of endophytic fungi specific to each group was relatively low, where Group 1 included 12 genera from 3 phyla; Group 2 included 11 genera from 3 phyla; Group 3 included 12 genera from 4 phyla; and Group 4 included 30 genera from 5 phyla. Among the endophytic bacteria unique to each group, Group 1, 2 and 4 contained 3, 3 and 2 genera with relatively high abundance, respectively, of which Group 1 included 32 genera from 5 phyla; Group 2 included 30 genera from 11 phyla; Group 3 included 21 genera from 10 phyla; and Group 4 included 55 genera from 13 phyla. Among the shared endophytes, the endophytic fungi with high abundance were *Aspergillus* and *Fusarium* of Ascomycota phylum and *Sebacinales* of Basidiomycota phylum, and the endophytic bacteria with high abundance were *Rhizobacter* and *Sphingomonas* of Proteobacteria phylum and *Arthrobacter* and *Rhodococcus* of Actinobacteria phylum. This showed that the number of unique endophytes in lithophytic Group 4 was higher than that of other arboreal groups.

## Discussion

Many microbial species including bacteria and fungi would interact with plants. This interaction could be symbiotic, mutualistic, parasitic or commensalistic (Bharadwaj et al. 2020). Endophytes exhibited complex diversity depending upon the different environmental conditions and this diversity could be exploited for the improvement of medicinal plant and the optimization of sustainable medicinal ingredients. The plant-endophyte partnerships are highly specific, and they own special recognition ways (Bharadwaj et al. 2020; Chen et al. 2021). This depends on a series of abiotic and biotic factors, such as the genotypes of plants and microbes, environmental conditions, and the dynamic network of interactions within the plant biome (Hardoim et al. 2015). Therefore, the diversity of endophytes in roots of lithophytic and arboreal *D. officinale* is stable and variable.

### The composition stability of endophytes in *D. officinale* at the phylum level

It has been found that endophytic fungi of *D. officinale* mainly include 57 genera of Basidiomycota, Ascomycota and Deuteromycotina, and endophytic bacteria mainly include 50 genera of Firmicutes, Proteobacteria, Actinobacteria, Bacteroidetes and Fusobacteria (Santoyo et al. 2016; Sarsaiya et al. 2020; Wang et al. 2022; Zhang et al. 2019). In this study, the endophytic fungi in the roots of *D. officinale* were mainly Basidiomycota and Ascomycota, and the endophytic bacteria were mainly Proteobacteria, Actinomycetes, Firmicutes, Bacteroides, Acidobacteria, etc. It can be seen that the endophytes of *D. officinale* were relatively stable at the phylum level composition.

### The composition stability and variability of endophytes in *D. officinale* at the genus level

The dominant endophytes in the roots of lithophytic type and arboreal types growing on different living trees were totally different in this study. For example, the dominant endophytes in the roots of lithophytic type were *Diplodia*, *Fusarium* and other fungal genera, *Pseudomonas*, *Bacillus* and other bacterial genera, respectively; the dominant endophytes of *D. officinale* growing on *Albizia* trees were fungal genera such as *Penicillium* and *Cladosporium*, and bacterial genera such as *Spirosoma*. Moreover, the endophytic fungi identified in *D. officinale* by Sarsaiya et al. (2020) were consistent with the biomarkers of Group 4, such as *Fusarium*, *Hypocreales*, *Sordariomycetes*.

The endophytic genera identified in this study were partly the same as those isolated and identified by predecessors, such as *Penicillium* and *Fusarium* in endophytic fungi (Liu C et al. 2017; Sarsaiya et al. 2020; Shi et al. 2018), *Pseudomonas* and *Bacillus* in endophytic bacteria (Shen et al. 2020). Predecessors found that *Fusarium*, *Glomerella*, and *Cladosporium* were the dominant genera of *D. officinale* (Liu W et al. 2017). In this study, it was found that the abundance and composition of dominant endophytes were different in the same epiphytic substrate in different habitats (Table 1, Figures 2, 5), indicating that environmental factors such as temperature, precipitation, and altitude had a greater impact on dominant endophytes, but for endophytic fungi and endophytic bacteria, the main environmental influences are different. In addition, previous studies have also found that altitude, temperature, and precipitation (Firrincieli et al. 2020; Hereme et al. 2020; Oita et al. 2021) were important environmental factors which affected the abundance and composition of endophytes, and endophytes could help plants adapt to different habitats (Suryanarayanan and Shaanker 2021). The endophytic fungi diversity of wild *D. officinale* was significantly higher than that of cultivated plants (Liu et al. 2020). In this study, the lithophytic *D. officinale* had higher diversity and more unique endophytes than arboreal types. At the same time, the proportion of Ascomycota in the samples was higher than that of Basidiomycota, which was consistent with previous studies (Liu W et al. 2017). Therefore, the composition of endophytes of *D. officinale* at the genus level had both stability and variability, which may be related to a variety of environmental factors.

### The specificity of the dominant endophytic genera in *D. officinale*

From the results of this study, it was clear that each group had its own unique dominant genera, which may have unique roles in helping *D. officinale* to take up nutrients, adapt to the environment, and synthesize secondary metabolites, such as *Penicillium* genus in Group 1, *Exophiala* genus in Group 4. Endophytic *Penicillium* had been reported to have multiple biological functions as biocatalysts, plant growth promoters, phytoremediators, and enzyme producers (Toghueo and Boyom 2020). Endophytic *Exophiala* genus may play a role in helping plants with heat stress tolerance (Khan et al. 2012).

In addition, pathogenic microbes such as *Fusarium* (Ma et al. 2013), *Pseudomonas* (Mansfield et al. 2012; Xin et al. 2018), and antagonistic microbes of pests or pathogens such as *Bacillus* (Fira et al. 2018), *Cladosporium* (Islam 2022) were among the dominant genera of *D. officinale*, reflecting the mutual check and balance of beneficial and pathogenic microbes.

In conclusion, we analyzed the endophyte diversity of arboreal and lithophytic *D. officinale* roots in the two kinds of habitats by high-throughput sequencing, revealing their diversity characteristics, abundance information, microbial biomarkers, dominant and special endophytes. Our results indicated that there was certain stability and variability in the composition of endophytes of arboreal and lithophytic *D. officinale* roots, as well as differences in diversity. It will be beneficial to use microbial resources to help the artificial cultivation or selection of *D. officinale*. In the future, we will screen microorganisms conducive to the synthesis of pharmacodynamic components for isolation, identification and functional characterization, so as to realize the development and utilization of biological inoculants.

## Data Availability

The data that support this study are available in National Microbiology Data Center at https://nmdc.cn/submit/dashboard/ [accession number: NMDCX0000108]. The other data that support this study are available in the article and accompanying supplementary material.

## Description of Supplementary Files

Supplementary data (Figure S1 and S2) associated with this article version, at https://doi.org/10.5511/plantbiotechnology.23.0208a.
