# Supplementary material for: Comparative analysis of endophyte diversity of Dendrobium officinale lived on rock and tree
Source: Plant Biotechnol (Tokyo). 2023 Jun 25;40(2):145–55. doi: 10.5511/plantbiotechnology.23.0208a (PMC10804140; doi:10.5511/plantbiotechnology.23.0208a)
Supplement: Supplementary Data [file plantbiotechnology-40-2-23.0208a-s001.pdf]

Supplementary Files Cladogram of endophyte biomarkers in *D. officinale roots*

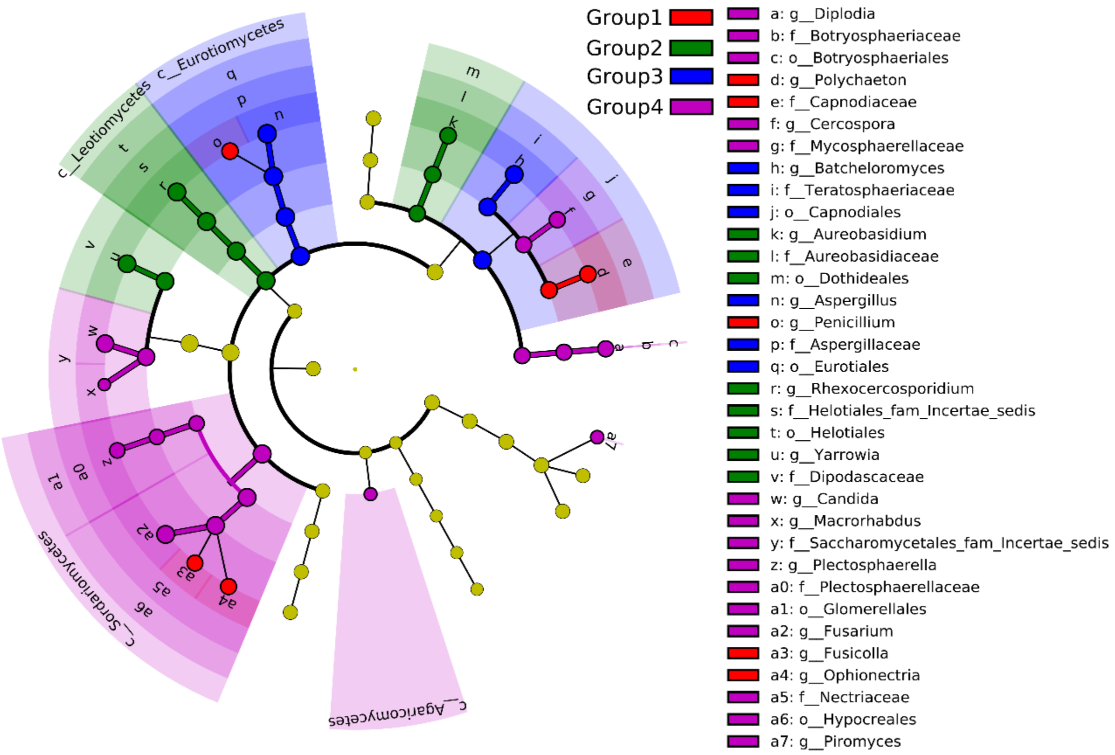

Figure S1 Cladogram of endophytic fungi biomarkers

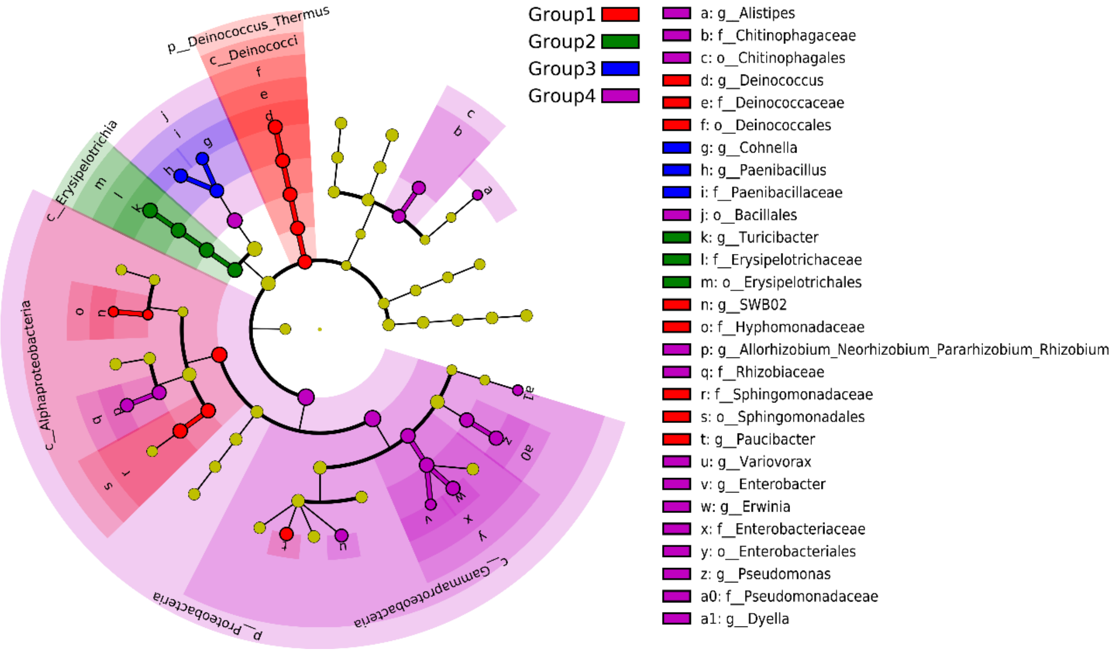

Figure S2 Cladogram of endophytic bacteria biomarkers
